# Challenges and Opportunities for Biomarkers of Clinical Response to AHSCT in Autoimmunity

**DOI:** 10.3389/fimmu.2018.00100

**Published:** 2018-02-02

**Authors:** Kristina M. Harris, Tingting Lu, Noha Lim, Laurence A. Turka

**Affiliations:** ^1^Immune Tolerance Network, Bethesda, MD, United States; ^2^Center for Transplantation Sciences, Massachusetts General Hospital, Boston, MA, United States

**Keywords:** autoimmunity, multiple sclerosis, immune cell reconstitution, biomarkers, immune tolerance, flow cytometry, T cells

## Abstract

Autoimmunity represents a broad category of diseases that involve a variety of organ targets and distinct autoantigens. For patients with autoimmune diseases who fail to respond to approved disease-modifying treatments, autologous hematopoietic stem cell transplantation (AHSCT) after high-dose immunosuppressive therapy provides an alternative strategy. Although more than 100 studies have been published on AHSCT efficacy in autoimmunity, the mechanisms that confer long-term disease remission as opposed to continued deterioration or disease reactivation remain to be determined. In a phase II clinical trial, high-dose immunosuppressive therapy combined with autologous CD34^+^ hematopoietic stem cell transplant in treatment-resistant, relapsing-remitting multiple sclerosis (RRMS) resulted in 69.2% of participants achieving long-term remission through 60 months follow-up. Flow cytometry data from the 24 transplanted participants in the high-dose immunosuppression and autologous stem cell transplantation for poor prognosis multiple sclerosis (HALT-MS) trial are presented to illustrate immune reconstitution out to 36 months in patients with aggressive RRMS treated with AHSCT and to highlight experimental challenges inherent in identifying biomarkers for relapse and long-term remission through 60 months follow-up. AHSCT induced changes in numbers of CD4 T cells and in the composition of CD4 and CD8 T cells that persisted through 36 months in participants who maintained disease remission through 60 months. However, changes in T cell phenotypes studied were unable to clearly discriminate durable remission from disease reactivation after AHSCT, possibly due to the small sample size, limited phenotypes evaluated in this real-time assay, and other limitations of the HALT-MS study population. Strategies and future opportunities for identifying biomarkers of clinical outcome to AHSCT in autoimmunity are also discussed.

## Introduction

Autologous hematopoietic stem cell transplantation (AHSCT) therapy utilizes immunoablation and immune reconstitution from hematopoietic progenitors. For more than two decades, AHSCT has been studied as a therapeutic approach for severe autoimmunity, including multiple sclerosis (MS), systemic sclerosis (SSc), systemic lupus erythematosus (SLE), Crohn’s disease, type 1 diabetes (T1D), and rheumatoid arthritis (RA) (1). The rationale for AHSCT for treating aggressive autoimmunity is that immunoablative therapy diminishes the pool of self-reactive immune cells and allows engrafted stem cells to generate a new, potentially self-tolerant, immune repertoire (2). Mechanistic studies have revealed qualitative changes in the reconstituted immune system after AHSCT that favor immunoregulation over pro-inflammatory signatures (2, 3). The tolerogenic properties of the reconstituted immune system likely result from coordinated interactions of various immune competent cells with regulatory potential. This dynamic and collective process of immune reprogramming is thought to underlie AHSCT’s mode of action.

Although more than 100 studies have been published, the mechanisms that confer long-term disease remission with AHSCT in autoimmunity, as opposed to limited short-term benefit and early disease recurrence, are not well understood. Restoration of regulatory immune networks may contribute to durable treatment; however, functional assessments of the reconstituted immune system compared to pretherapy profiles are needed to understand how AHSCT limits or controls autoreactive lymphocytes during and after repopulation. Currently, it is unclear whether relapse after AHSCT is driven by residual autoreactive memory cells that escaped depletion and are resistant to regulatory mechanisms in the renewed immune system or to re-emergence of a *de novo* autoreactive population, possibly reflecting a genetic predisposition to disease. In this perspective, we present data from the high-dose immunosuppression and autologous stem cell transplantation for poor prognosis multiple sclerosis (HALT-MS) trial to illustrate past and present approaches to address this question and discuss experimental challenges and strategies for identifying the biomarkers of clinical response to AHSCT in autoimmunity.

## Immune Tolerance Network (ITN) HALT-MS Trial Experience

High-dose immunosuppression and autologous stem cell transplantation for poor prognosis MS was a phase II clinical trial conducted by the ITN that investigated the efficacy of AHSCT in treatment-resistant patients with relapsing-remitting multiple sclerosis (RRMS) (4). Twenty-four participants underwent AHSCT and were evaluated through 60 months posttransplant for event-free survival, defined as survival without death or disease activity. Progression-free survival, clinical relapse-free survival, and magnetic resonance imaging (MRI) activity-free survival were 91.3, 86.9, and 86.3%, respectively, indicating that AHSCT without maintenance disease-modifying therapy was effective for inducing durable remissions of active RRMS for at least 5 years (4). The primary mechanistic objectives for the HALT-MS trial were to determine the impact of AHSCT on the diversity of T cell receptor (TCR) repertoires in reconstituted peripheral blood and intrathecal compartments and to assess the treatment effect on pro-inflammatory versus regulatory T cell phenotypes in peripheral blood.

Here, we present flow cytometry data from HALT-MS to demonstrate the characteristics of immune reconstitution in RRMS patients through 36 months post-AHSCT and to highlight potential confounders that interfere with identification of biomarkers for relapse and long-term remission. Of the 24 transplanted participants in HALT-MS, 3 experienced clinical relapse, 2 showed disease progression by increased Expanded Disability Status Scale, and 2 had increased MRI through 60 months follow-up. Results are displayed as mean values for the long-term remission group (*n* = 15–17) with individual lines for the seven participants who experienced disease reactivation at different times through 60 months follow-up to help illustrate the obstacles these variances impose on our biomarker efforts. We hypothesized that favorable changes in the balance of pro-inflammatory and regulatory/naive/hyporesponsive T cell phenotypes in reconstituted peripheral blood would be associated with long-term remission after AHSCT. Paired statistical comparisons pretherapy to posttherapy were restricted to the long-term remission group because of the sample size and heterogeneity of the group that experienced disease reactivation during the 60 months post-AHSCT follow-up.

**Figure 1:**
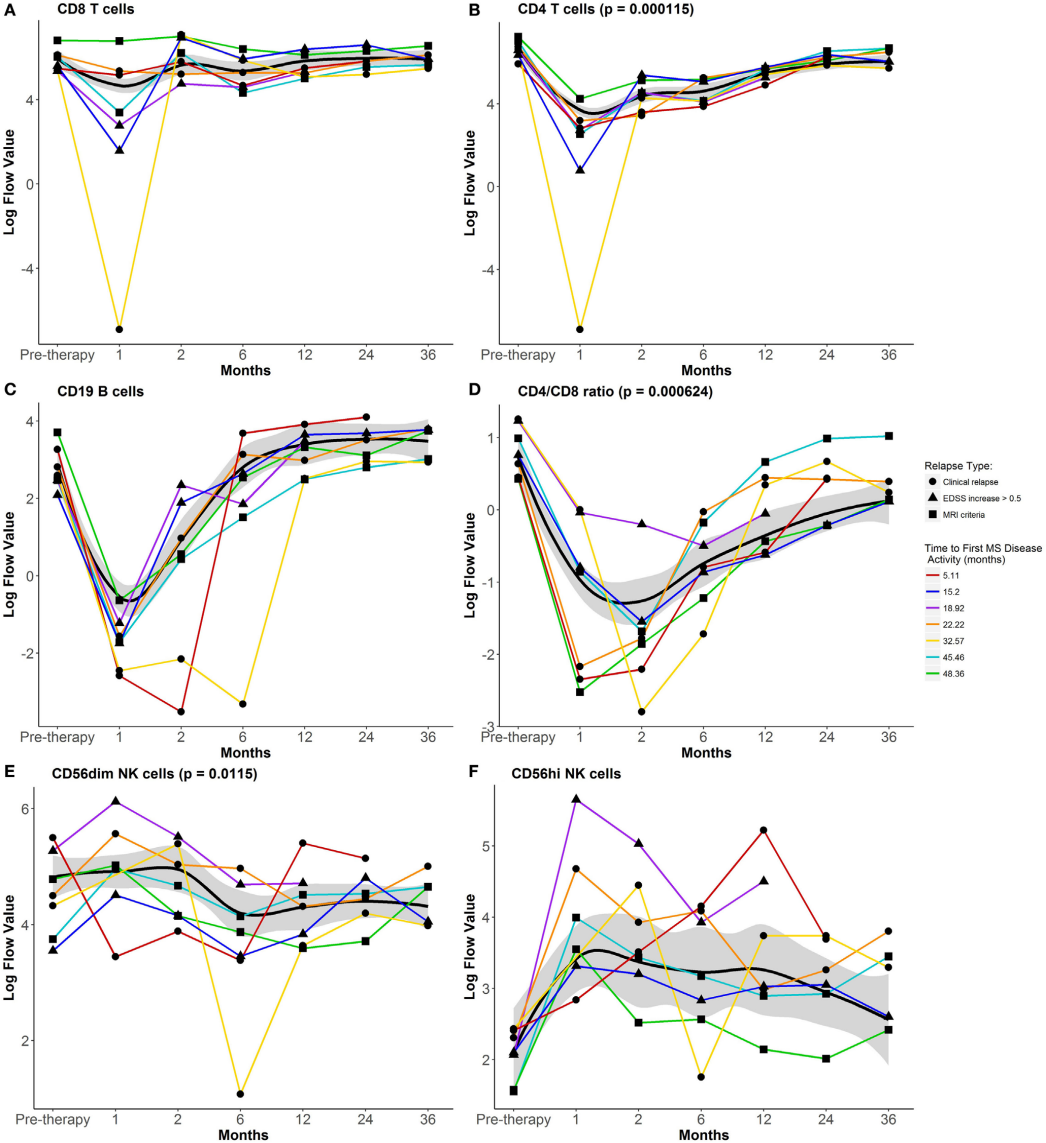
Impact of autologous hematopoietic stem cell transplantation (AHSCT) on numbers of circulating lymphocyte populations through 36 months follow-up. Absolute cell numbers per microliter of whole blood were analyzed for **(A)** CD8 T cells, **(B)** CD4 T cells, **(C)** CD19 B cells, **(E)** CD56^dim^ NK cells, and **(F)** CD56^hi^ NK cells. **(D)** CD4/CD8 ratios were calculated from absolute cell numbers **(A,B)**. Flow data were plotted after log transformation for normalization of these variables. Data shown are mean values for the group that maintained remission through 60 months post-AHSCT. The black line represents the Loess Regression fitted curve with a span = 0.7, and its 95% confidence band colored in gray. Paired *t*-test was used to examine persistent changes at 36 months from pretherapy numbers within the group that maintained remission through 60 months post-AHSCT (n = 15). CD4 T cells, CD4/CD8 T cell ratio, and CD56^dim^ NK cells were reduced from pretherapy numbers at 36 months post-AHSCT. Individual lines for the seven participants who experienced disease reactivation prior to 60 months post-AHSCT are plotted using different symbols to indicate the type of disease activity and different colored lines to indicate the time to first multiple sclerosis (MS) disease activity. For additional details including flow cytometry data without log-transformation, see https://www.itntrialshare.org/HALTMS_fimmu_fig1.url.

**Figure 2:**
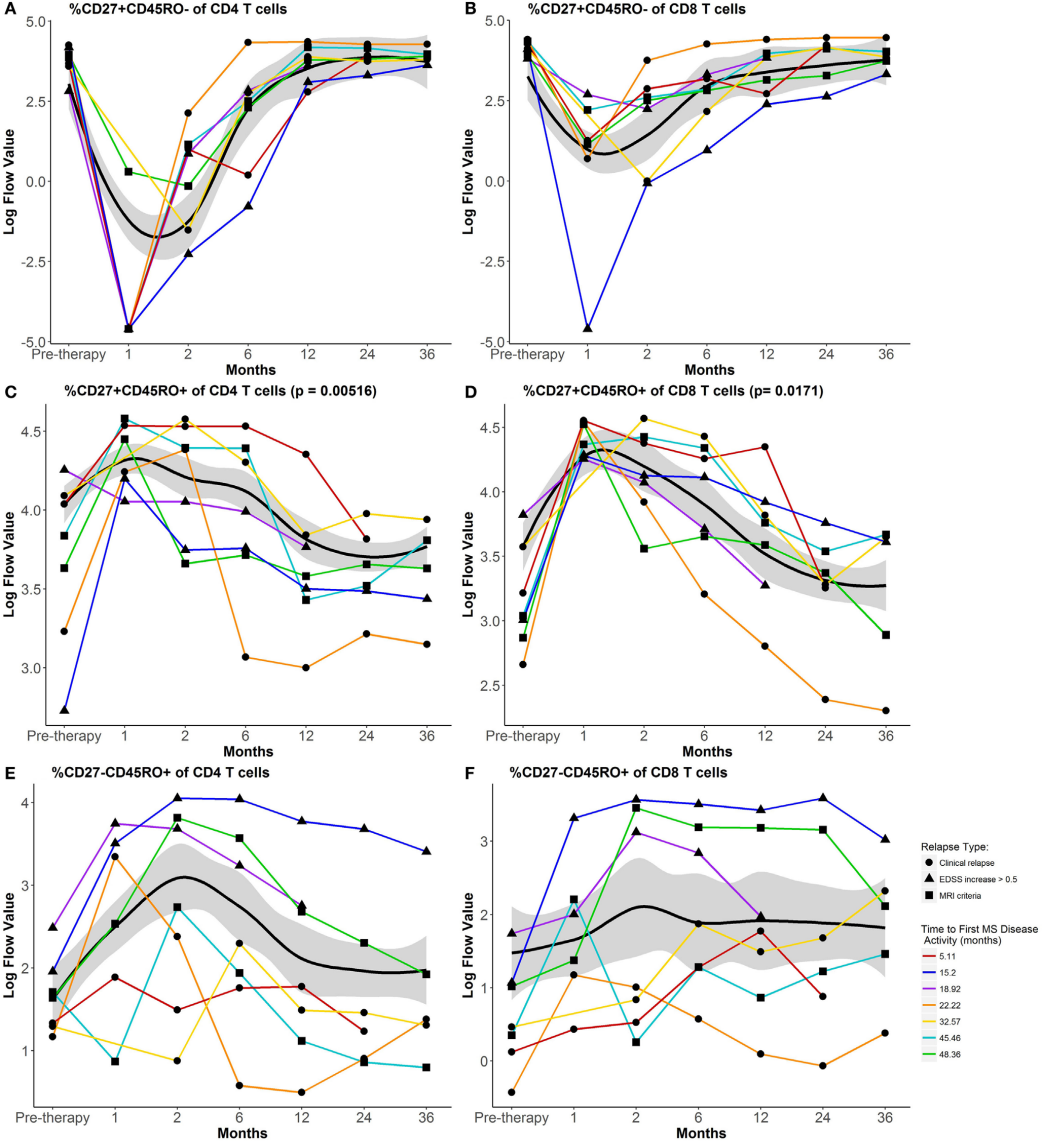

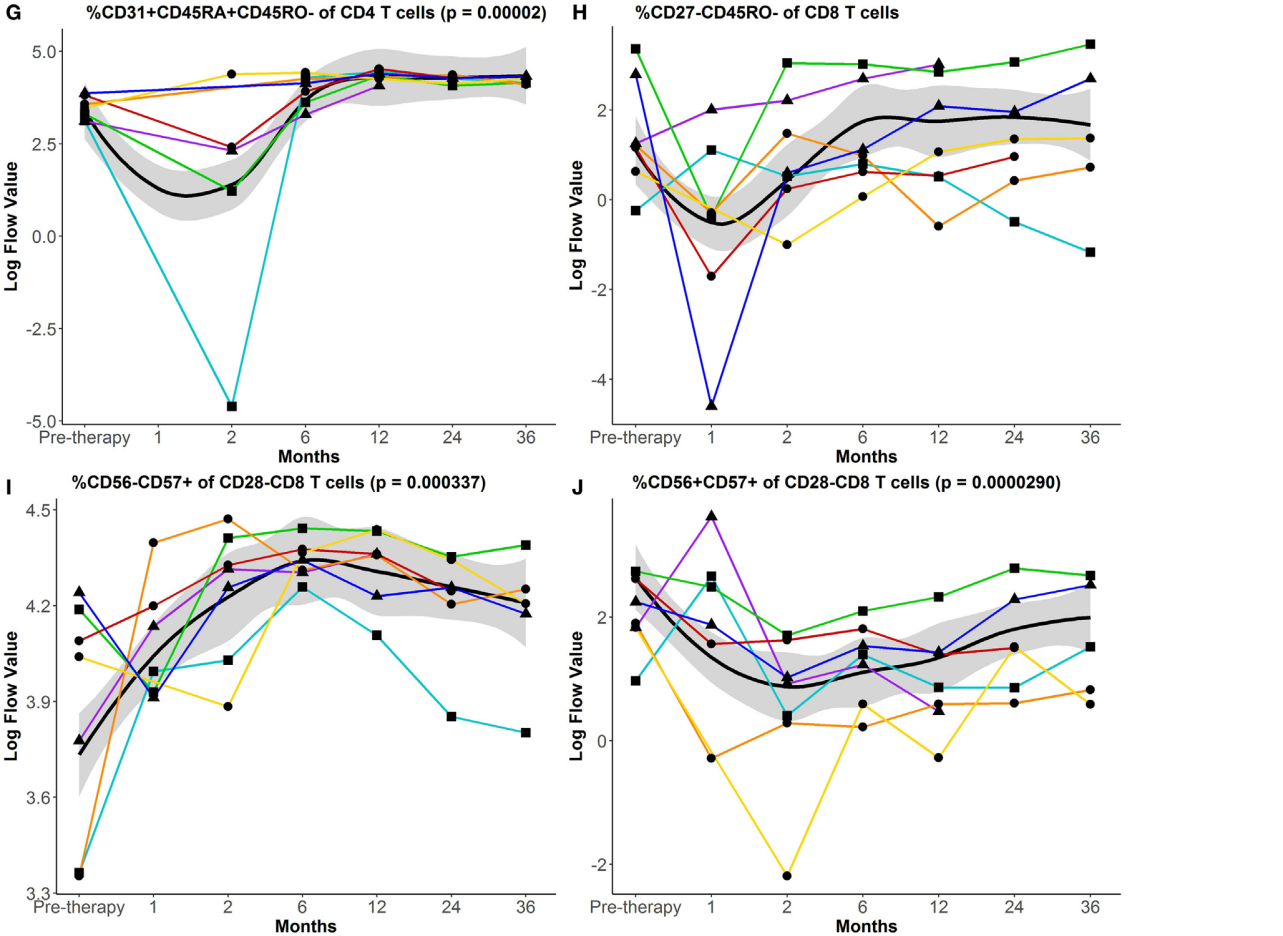
Impact of autologous hematopoietic stem cell transplantation (AHSCT) on the composition of circulating CD4 and CD8 T cells through 36 months follow-up. Percentages of CD27^+^CD45RO^−^ naive CD4 **(A)** and CD8 **(B)** T cells, CD27^+^CD45RO^+^ central memory CD4 **(C)** and CD8 **(D)** T cells, CD27^−^CD45RO^+^ effector memory CD4 **(E)** and CD8 **(F)** T cells, **(G)** CD31^+^CD45RA^+^CD45RO^−^ CD4 recent thymic emigrants (RTEs), **(H)** CD27^−^CD45RO^−^ long-term memory CD8 T cells, **(I)** CD56^−^CD57^+^CD28^−^ CD8 T cells, and **(J)** CD56^+^CD57^+^CD28^−^ CD8 T cells. Flow data were plotted after log transformation for normalization of these variables. Data shown are mean values for the group that maintained remission through 60 months post-AHSCT. The black line represents the Loess Regression fitted curve with a span = 0.7, and its 95% confidence band colored in gray. Paired *t*-test was used to examine sustained changes at 36 months from pretherapy numbers within the group that maintained remission through 60 months post-AHSCT (n = 15). AHSCT induced persistent changes in relative proportions of central memory and RTE phenotypes in reconstituted CD4 T cells. In reconstituted CD8 T cells, sustained alterations in proportions of central memory, CD56^−^CD57^+^CD28^−^ (senescent) and CD56^+^CD57^+^CD28^−^ (cytotoxic) phenotypes were observed. Individual lines for the seven participants who experienced disease reactivation before 60 months post-AHSCT are plotted using different symbols to indicate the type of disease activity and different colored lines to indicate the time to first multiple sclerosis (MS) disease activity. For additional details including flow cytometry data without log-transformation, see https://www.itntrialshare.org/HALTMS_fimmu_fig2.url.

Most differences in circulating lymphocytes occurred early after AHSCT at 1–2 months. Absolute numbers of CD8 and CD4 T cells and B cells diminished, while CD56^hi^ precursor NK cells expanded after AHSCT (Figure 1). Numbers of CD8 T cells and B cells returned between 2 and 6 months post-AHSCT (Figures 1A,C), whereas CD4 T cells and CD4/CD8 ratios remained significantly decreased at 36 months post-AHSCT compared to pretherapy (Figures 1B,D). Numbers of CD56dim cytotoxic NK cells declined at 6 months post-AHSCT and remained reduced from pretherapy at 36 months. Early during reconstitution, CD4 and CD8 T cells reflected a bias toward memory phenotypes with reduced proportions of CD27^+^CD45RO^−^ naive cells (Figures 2A,B). Following their early increase, CD27^+^CD45RO^+^ central memory cells decreased and stayed significantly lower than pretherapy proportions at 36 months (Figures 2C,D). CD27^−^CD45RO^+^ effector memory cells transiently increased early post-AHSCT (Figure 2E,F). CD31^+^CD45RA^+^CD45RO^−^ CD4 recent thymic emigrants (RTEs) increased after their initial decline and remained significantly elevated at 36 months post-AHSCT, indicative of thymic renewal (Figure 2G). CD27^−^CD45RO^−^ long-term memory CD8 T cells transiently diminished and reached pretherapy proportions at 36 months post-AHSCT (Figure 2H). A relative expansion of potentially senescent (5) CD28^−^CD56^−^CD57^+^ CD8 T cells (Figure 2I) was observed after AHSCT at the expense of cytotoxic CD28^−^CD56^+^CD57^+^ CD8 T cells (Figure 2J). These results are consistent with previous immunophenotyping studies in patients with MS and other autoimmune diseases treated with AHSCT (6–11).

Potential T cell biomarkers of response to AHSCT have been reported in MS and T1D (6, 7), including the expansion of regulatory CD4 (Foxp3^+^CD127^lo^) and CD8 (CD28^−^CD57^+^ or PD-1^+^) T cells. However, T cell phenotypes that clearly discriminate disease reactivation from long-term remission post-AHSCT have not been identified in the immunophenotyping studies presented here or previously published for HALT-MS (10). Overall reconstitution patterns appeared similar for participants who did and did not achieve remission through 60 months post-AHSCT. While a few dramatic deviations from the mean of the long-term remission group were observed for individuals who did not maintain remission, no consistent changes in cell subtypes studied characterized all or any of the three categories of disease reactivation. These changes could reflect true biology or could be spurious findings unrelated to disease activity or adverse events. Two of the seven participants were in remission at the time of this analysis and relapsed by MRI activity later on; therefore, it is possible that relevant biomarkers had not yet re-emerged. Interpretation is limited by the sample size and various times to disease reactivation, particularly since specimens were not collected temporal to disease activity before rescue medication was given. Expansion of regulatory CD4 T cells (Foxp3^+^CD25^hi^, CD25^hi^CD127^lo^) has been reported in AHSCT trials in MS, SSc, SLE, and T1D (6, 7, 12–14); however, an equivalent phenotype was not analyzed when this “real-time” flow analysis was initiated at the start of the HALT-MS trial. This highlights one of the major limitations of real-time assays for biomarker research; they are often outdated by the end of the trial. Highly sophisticated immunophenotyping studies are in progress to assess reconstitution of T and B cell phenotypes relevant to MS and autoimmunity post-AHSCT; however, our response to biomarker efforts will still be confounded by the small sample size and suboptimal collection schedule of participants who experienced disease reactivation after AHSCT during the 60 months follow-up in HALT-MS. These challenges and others are discussed below.

## Challenges of Biomarker Discovery for Clinical Response to AHSCT

### Clinical Trial Design and Disease Heterogeneity

Nearly all studies evaluating AHSCT in autoimmunity have been open-label, single-arm, observational phase I/II trials (1, 2). Although highly efficacious, the nature of these trials imposes challenges on biomarker research. These include small numbers of transplanted participants, fewer subjects in potentially unbalanced and subjective binary outcome groups, and the lack of appropriate controls. Heterogeneity within a given autoimmune disease can manifest as a spectra of disease severity, duration, clinical manifestations, and tissue targets. Biological diversity and treatment history of a study population can also influence participant responses at each step of the transplantation procedure, including stem cell mobilization, conditioning efficacy, stem cell engraftment, and immune reconstitution. The timing and type of prior immunotherapy can alter the immunological profile pretherapy with implications on biomarkers that predict or correlate with response to AHSCT. Inconsistencies between transplantation protocols can influence the range, degree, and kinetics of immune cell depletion and reconstitution. For example, autoimmune activity may persist if depletion of autoreactive lymphocytes is incomplete or re-establish with the graft, depending on the conditioning regimen and grafted stem cells (i.e., CD34^+^ selected or not) (15). Some of these interindividual variables can and should be mitigated during protocol development with careful planning of inclusion/exclusion criteria for the study.

### Specimen Collection

Since the primary objective of AHSCT is to achieve durable remission of autoimmunity without immunosuppressive agents, a long-term follow-up period is required for the assessment of clinical and scientific goals. This provides researchers the opportunity to monitor the dynamic process of immune reconstitution for biomarker discovery. It also magnifies potential variances between biomarker studies as standardized schedules for specimen collection post-AHSCT were lacking. Guidelines for immune monitoring and biostorage of AHSCT-treated autoimmune patient specimens have been published to provide a consistent framework for future biomarker research (16). Specimens should be collected at two time points pretherapy, at regular intervals post-AHSCT, and proximal to the first disease-related event prior to disease-modifying treatment. The latter is essential for identifying changes in biomarkers associated with durable versus short-term remission to AHSCT.

Serial samples of whole blood, peripheral blood mononuclear cells (PBMCs), plasma, serum, saliva, and urine specimens can be readily obtained in sufficient volumes for biomarker studies of AHSCT in autoimmunity. In contrast, sequential sampling of disease-relevant tissues may not be possible or may be limited due to ethical constraints and the practicalities of trial conduct. Preanalytical variations can be introduced by sample types, timing, technique, and collection devise; handling and storage conditions, including stabilizing agents, temperature, duration, and freeze–thaw cycles; and documentation of specimen data (17). Since few patients are transplanted at each site in multicenter trials, these variables can adversely affect overall findings and reproducibility of biomarker studies. Therefore, standard and optimized protocols must be followed for each step of specimen collection. Centralized processing and testing of high-quality, banked specimens at designated, well-qualified laboratories is strongly recommended. Cryopreserved specimens are particularly useful because they permit focused design and execution of biomarker studies at the end of the trial, when all end points have been identified. Banked specimens allow simultaneous testing of all visits from a participant using state-of-the-art technologies in a single laboratory. This helps minimize interassay deviations, avoids testing immune parameters and technologies that may become obsolete during the trial, and eliminates interlaboratory variability. All of which can confound detection of changes in biomarkers that are rare such as autoreactive and regulatory lymphocytes. Although banked specimens are preferred for mechanistic studies, it is beneficial to perform a reliable full blood count analysis on fresh specimens when feasible to enumerate major leukocyte populations for interpreting immunological changes induced by AHSCT in banked specimens.

### Experimental Design

Mechanistic studies of AHSCT in autoimmunity have focused on disease-specific autoimmune parameters in the context of general immune reconstitution using immunophenotyping and transcriptional analyses. Despite rapidly evolving technologies and variation between cohorts, this global approach has revealed similar patterns of immune reconstitution and qualitative changes in PBMC in different autoimmune diseases (3, 18–20). This supports the hypothesis that AHSCT reprograms the self-destructive immune system toward a tolerant state; however, more sophisticated functional studies and analytical approaches are required for discovery and validation of biomarkers of response to AHSCT.

Different types of autoimmunity are associated with distinct immunological signatures; therefore, biomarker(s) of response to AHSCT may be disease specific and require different assays. The prevailing view is that both B cells and T cells are important in SSc, SLE, and MS, whereas the role of B cells in T1D is not clear (2). Previous studies indicated that myelin-specific CD4 T cell responses are initially limited in MS patients after AHSCT, but return to pretherapy levels after immune reconstitution (21, 22). It is not evident whether these autoreactive CD4 T cells re-emerged through incomplete immune ablation or were generated *de novo* and if they are even relevant to disease. Reliable *ex vivo* assays for phenotyping and sorting disease-relevant central nervous system (CNS) antigen-reactive T cells for molecular assays pretherapy to posttherapy are highly desirable because they could determine whether functional differences of reconstituted T cells are associated with clinical response. However, validated assays for MS clinical trials that do not require *in vitro* manipulation are still in development, in part because CNS antigen-reactive T cells are rare in blood of MS patients, and disease-relevant CNS antigens need to be clarified. This approach could be used in other diseases where the autoantigen is known, and *ex vivo* tetramer reagents are available, such as in T1D (7).

In the absence of a validated assay for assessing autoreactive T cells in HALT-MS, we used TCR repertoire analysis of CD4 and CD8 T cells in blood and the cellular fraction of cerebrospinal fluid (CSF) to better understand how AHSCT shapes adaptive immunity in the reconstituted immune system. CSF is the compartment in closest proximity to the CNS parenchyma that might reflect immune pathology in MS. The impact of AHSCT on TCR repertoire diversity was investigated during the HALT-MS trial, and distinct effects on circulating CD4 and CD8 T cells were identified. The majority of CD4 TCR clones arose *de novo*, and there was expansion of pre-existing CD8 clones through 12 months post-AHSCT (23). Reconstitution of a new and diverse TCR repertoire in blood has been reported in other trials of AHSCT in autoimmunity; however, it is difficult to compare with our findings because of a number of technical differences (11, 24, 25). Additional TCRβ sequencing analyses are in progress to evaluate the impact of AHSCT on the pre-existing TCR repertoire in CSF and to determine whether AHSCT changes the pattern of clonal sharing between CSF and blood in HALT-MS.

### Analytical Approach and Biomarker Validation

Overwhelming amounts of mechanistic data can now easily be generated and integrated with relevant clinical data for hypothesis-based and unbiased biomarker discovery approaches. This presents a major challenge for determining the right data sets to include and the appropriate statistical models needed to address key scientific questions (26). Different data sets and analytical approaches are necessary for addressing the two primary aims shared by all mechanistic studies of AHSCT, which include identification of biomarkers that predict, and those that correlate with, response outcomes. Biomarkers that predict outcome can be used to stratify patient populations for improved efficacy of AHSCT. However biomarkers refractory to, or induced by, AHSCT that correlate with poor outcome may help identify therapeutic targets for more durable remission with sequential administration of approved agents. For example, a small study by Capobianco et al. indicated that natalizumab after AHSCT could be an effective strategy for RRMS patients who continue to deteriorate after AHSCT therapy (27).

Before putative biomarkers that correlate with clinical response can help guide clinical practice, they must be validated and shown to be clinically meaningful. This is the biggest challenge for biomarker efforts because well-matched cohorts may not be available due to inconsistencies in trial designs and specimen collection (17) or in response to safety concerns like high morbidity in the T1D trial of AHSCT (28). Validation cohorts should involve prospective, controlled, randomized trials with standardized specimen collection, reliable assays, bioinformatics, and data sharing.

## Opportunities for Biomarkers of Response to AHSCT

High-dose immunosuppression and autologous stem cell transplantation for poor prognosis MS demonstrated that it is possible to achieve durable remission in patients with treatment-resistant RRMS using AHSCT therapy (4). We speculate that a combination of persistent changes in T cell numbers and composition that support increased thymic output, TCR repertoire renewal, and immune senescence and/or regulation over central memory or cytotoxicity contribute to long-term remission in HALT-MS. Still, functional and transcriptional studies of autoreactive, pro-inflammatory, and tolerogenic T and B cell subsets collected at optimal time points from sufficient numbers of participants in long-term remission and disease reactivation groups are needed to show that AHSCT induces qualitative changes in favor of immune tolerance that persist in patients achieving durable remission. In addition, TCR/B cell receptor sequencing in CSF and blood are necessary to identify clonotypes associated with disease reactivation and to determine whether renewal/diversification of the reconstituted adaptive immune system contributes to long-term remission post-AHSCT. HALT-MS can provide clues about some of these important questions; however, validation of putative response biomarkers will require larger patient numbers, independent cohorts, and appropriate controls.

The success of the HALT-MS trial has provided the foundation for a clinical trial in the development by the National Institutes of Health and the ITN that will compare AHSCT with best available approved therapy in the treatment of RRMS. This trial presents a unique opportunity to follow-up on TCR repertoire and immunophenotyping studies from HALT-MS in a larger, controlled study. The primary mechanistic objective of the follow-up trial is to understand the mechanisms that distinguish AHSCT from the high-efficacy approved agents in the control arm. Quality specimens from peripheral blood and CSF will be interrogated using state-of-the-art technologies that become available and validated for analysis of primary end point outcomes to corroborate proposed and discover new, biomarkers of response to AHSCT compared to high-efficacy disease-modifying therapies. This knowledge will confirm the treatment rationale and help refine future protocols, so patients with aggressive RRMS can achieve durable remission in the absence of ongoing immunosuppression.

In summary, HALT-MS uncovered a number of challenges for identifying biomarkers of clinical response to AHSCT, many of which can be avoided in future trials through thoughtful trial design, specimen collection, experimental planning, bioinformatics, and data sharing. To achieve this, the ITN brings together a multidisciplinary team of clinicians, regulatory officials, research scientists, and biostatisticians through partnerships between academia, government, industry, and other research consortia. Data sharing is available through ITN TrialShare,^1^ an online resource that allows users open access to our clinical trial and mechanistic data for reuse and independent analysis to provide new concepts or insights that expand our knowledge of AHSCT in RRMS and beyond (29).

## Materials and Methods

### HALT-MS Subjects

Clinical data from the HALT-MS participants have been reported previously (4) and are available through ITN TrialShare.^2^

### Flow Cytometry

By using a stain-lyse method, peripheral blood cells shipped ambient overnight to the ITN Flow Cytometry Core at RPCI were stained with 5-color monoclonal antibody panels using anti-human CD3-PECy7, CD4-PERCP, CD4-APC, CD8-PERCP, CD31-FITC, CD45RA-APC, CD45RO-PE, CD27-FITC, CD28-APC, CD57-FITC, CD56-PE, CD19-PECy7 (all from BD Biosciences). Blinded samples were acquired on a Canto A flow cytometer (BD Biosciences), then gated, and analyzed using FlowJo Software (Tree Star Inc.).

### Statistical Considerations

Flow data were log-transformed for statistical analysis because they were not normally distributed. Paired *t*-test was used to compare changes from baseline at 36 months within the durable remission group. Analyses were performed in SAS 9.4 (SAS Institute, Cary, NC, USA), and graphics were plotted in R Version 3.4.1 (30).

## Data and Materials Availability

Data sets for these analyses are accessible through ITN Trial Share, a public website managed by the Immune Tolerance Network (www.itntrialshare.org/HALTMS_fimmu.url).

## Ethics Statement

The HALT-MS study [a Phase II Study of High-Dose Immunosuppressive Therapy (HDIT) Using Carmustine, Etoposide, Cytarabine and Melphalan (BEAM) + Thymoglobulin, and Autologous CD34+ Hematopoietic Stem Cell Transplant (HCT) for the Treatment of Poor Prognosis Multiple Sclerosis] was sponsored by NIAID and conducted by the ITN (ITN033AI) (ClinicalTrials.gov NCT00288626). This study was carried out in accordance with the recommendations of NIAID with written informed consent from all subjects. All subjects gave written informed consent in accordance with the Declaration of Helsinki. The protocol was approved by the by the IRB at each of the clinical sites.

## Author Contributions

LT contributed to concept development and experimental design. NL collected data. NL and TL helped with data analysis and visualization. KH interpreted the data and wrote the manuscript. All authors made contributions to the final manuscript prior to submission.

## Conflict of Interest Statement

The authors declare that the research was conducted in the absence of any commercial or financial relationships that could be construed as a potential conflict of interest.

## Footnotes

^1^www.itntrialshare.org.

^2^https://www.itntrialshare.org/study/Studies/ITN033AI5YR/Study%20Data/dataset.view?datasetId=502.

## Abbreviations

AHSCT, autologous hematopoietic stem cell transplantation; RRMS, relapsing-remitting multiple sclerosis; HALT-MS, high-dose immunosuppression and autologous stem cell transplantation for poor prognosis multiple sclerosis; SSc, systemic sclerosis; SLE, systemic lupus erythematosus; T1D, type 1 diabetes; NIH, National Institutes of Health; ITN, immune tolerance network; MRI, magnetic resonance imaging; EDSS, Expanded Disability Status Scale; TCR, T cell receptor; BCR, B cell receptor; RTE, recent thymic emigrants; PBMC, peripheral blood mononuclear cell; CNS, central nervous system; CSF, cerebrospinal fluid.
